# Chromosome-scale genome assembly and annotation of the white star apple (*Gambeya albida*)

**DOI:** 10.1038/s41597-026-08002-8

**Published:** 2026-08-04

**Authors:** Michael Landi, Sadik Muzemil, Adedapo O. Adediji, Laurah Nyasita Ondari, Kilsi Borgbara, Acclaim Moila, Adewale G. Awoyemi, Yedomon Ange Bovys Zoclanclounon, Tolulope R. Oladimeji, Ademola D. Ajayi, Ayodele O. Majekodunmi, ThankGod Echezona Ebenezer, Andreas Gisel, Lukman A. Aroworamimo

**Affiliations:** 1 Swedish University of Agricultural Sciences, Uppsala, Sweden; 2 International Institute of Tropical Agriculture, Nairobi, Kenya; 3 University of Warwick, Coventry, UK; 4 University of Liverpool, Liverpool, UK; 5 Pan African University Life and Earth Sciences Institute, University of Ibadan, Ibadan, Nigeria; 6 Inqaba Biotec, Ibadan, Nigeria; 7 Inqaba Biotec, Pretoria, South Africa; 8 International Institute of Tropical Agriculture, Ibadan, Nigeria; 9 Rothamsted Research, Harpenden, UK; 10 Minnesota State University, Mankato, USA; 11 Malimbe Foundation, Ibadan, Nigeria; 12 Ajisefini Consulting, Abuja, Nigeria; 13 EMBL-European Bioinformatics Institute (EMBL-EBI), Hinxton, United Kingdom; 14 African BioGenome Project (AfricaBP), Juja, Kenya; 15 African BioGenome Project (AfricaBP), Cambridge, United Kingdom; 16 Institute for Biomedical Technologies, CNR, Bari, Italy

## Abstract

White star apple (*Gambeya albida*) is native to the lowland rainforests of Central, East, and West Africa. This species is highly valued for its nutritious fruits and offers medicinal, socio-cultural, and economic benefits. In West Africa, it contributes to food security for rural and urban communities alike. However, no genomic resources are available to untap the agronomic and medicinal traits of the white star apple. Here, we present its first chromosome-scale genome, generated using PacBio HiFi and Omni-C sequencing. The white star apple genome is highly homozygous, and we assembled 98.9% of the estimated haploid genome size (822 Mbp) into 13 pseudochromosomes. It has a base-level accuracy (QV) of 58.14, an N50 of 57 Mbp, and 97.5% BUSCO completeness, representing a reference-quality assembly. About 58.5% of the genome constitutes repetitive sequences, and ab initio gene prediction identified 33,602 gene models. This reference-quality genome of the white star apple will serve as a valuable resource to enhance our understanding of its nutritional and pharmacological traits and facilitate improvement research.

## Background & Summary

White star apple (*Gambeya albida* (G. Don) Aubrév. & Pellegr., syn. *Chrysophyllum albidum* G. Don) is a woody plant species belonging to the family Sapotaceae, with its native range across the lowland rainforests of Central, East, and West Africa^1^. Commonly called Agbalumo (Yoruba), Udara (Igbo), and Agwaliba (Hausa) in Nigeria and Alasa in Ghana^2^, it can grow up to 45 m in height, with mature girth varying from 1.5 to 2 m^3^. The fruit is five-celled, enclosing five brown, bean-shaped seeds within a yellowish, pleasantly acidic pulp. These seeds are shiny when ripe and are distinctively arranged in a star pattern inside the fruits, hence the tree’s unique name.

The tree is indigenous to West and Central Africa with numerous nutritional, medicinal, socio-cultural, and economic benefits^4,5^. It plays a crucial role in food security, particularly in rural and urban areas of West Africa, serving as a vital food source and snack. Its fruit is a significant contributor to the diets of many communities, especially during its peak fruiting season from December to March^2^. The fleshy pulp of the fruit is highly relished by both young and old. Beyond direct consumption, the fruit is used to produce various food products, including stewed fruit, marmalade, syrup, and soft drinks, highlighting its versatility in food processing. Nutritionally, the white star apple fruit is rich in ascorbic acid, alkaloids, tannins, carbohydrates, crude fibre, lipids, potassium, and calcium^6–8^. For example, Amusa et al.^9^ found that white star apple fruits have a higher vitamin C content than oranges and 10 times that of guava or cashew. Its proximate analysis has shown the presence of moisture (73.33%), ash (2.64%), fiber (3.61%), protein (16.99%), and vitamins A, B1, and B2^10^. The presence of pectin, polyphenols, and various minerals further enhances its nutritional value, with potential for detoxification and reducing sugar absorption^11^. Studies have also indicated that the nutritional content can vary between sweet and sour fruits, though lipid and crude fiber values remain similar^12^. This rich nutritional profile underscores its importance in combating malnutrition and enhancing dietary diversity in regions where it is consumed^6^.

Traditional African medicine widely utilizes different parts of the white star apple for treating a wide range of ailments. Every part of the plant, including its leaf, seed, pulp, peel, bark, and root, is essentially considered valuable because of its anti-nociceptive, anti-inflammatory, and antioxidant properties^13,14^. Extracts from these various parts have demonstrated good antimicrobial and antifungal activities. For example, Eleagnine, an alkaloid isolated from white star apple seed cotyledon, has been found to exhibit these properties^15^. The fruit’s rich sources of natural antioxidants have been established to promote health by acting against oxidative stress-related diseases such as diabetes, cancer, and coronary heart diseases^16^. The plant’s therapeutic value is attributed to its rich phytochemical composition, including alkaloids, tannins, flavonoids, phenols, anthocyanins, and proanthocyanidins. In addition, the socio-economic usefulness of the white star apple has been reported in several studies^17–19^, highlighting its high value as a tree of support, financial profitability, and monetary returns, especially for African rural dwellers. The fruit also contains anacardic acid, industrially used for wood protection and as a resin source, while the seeds are a source of oil used for diverse purposes and for local games^20^.

The numerous uses of white star apple in tropical Africa have led to its unsustainable exploitation, which is the greatest threat to this species, combined with other factors such as habitat loss, deforestation, urbanisation, and climate change^21^. This has led to its classification as a ‘Near Threatened’ species by the International Union for Conservation of Nature^1^. Thus, conservation efforts aimed at reviving the declining populations of the white star apple are necessary^22^.

Here, we present a high-quality chromosome-level genome of the white star apple. In addition, we report one of the first plant genome sequenced and assembled 100% locally in Africa, utilising highly accurate PacBio HiFi and Omni-C chromatin conformation data. As of 2022, of the 798 plant species sequenced globally, only 20 are endemic to Africa, and none of these were sequenced locally in Africa^23^. Previous plant species sequenced in Africa either utilised Oxford Nanopore Technologies (ONT) and chromosome conformation capture (Hi-C) sequencing, lablab (*Lablab purpureus*), and African yam bean (*Sphenostylis stenocarpa*)^24,25^. This white star apple genome will facilitate breeding and conservation efforts, enhance our understanding of key metabolic pathways and the underlying genes for nutritional and pharmacological applications, and showcase the potential for genomic studies to be fully implemented in Africa.

## Methods

### Ethics statement

Sample collection procedure, including processing and handling of the plant used in this study, followed the Collection Policy for tree propagation at the International Institute of Tropical Agriculture (IITA), which was approved by the Manager of the IITA Forest Center. Approval for the collection, handling, and international transfer of plant genetic material was granted via a legally binding Material Transfer Agreement (MTA) executed between the Federal Ministry of Environment of the Federal Republic of Nigeria and Inqaba Biotec West Africa Ltd. These were guided by the African BioGenome Project (AfricaBP) Ethical, Legal, and Social Issues Subcommittee’s practical guide on accessing and sharing biological materials and data^26^, enabling the later-stage processes that were carried out in accordance with local biodiversity, biosafety, and access-and-benefit-sharing regulations of the Federal Republic of Nigeria, including adherence to the Nagoya Protocol. The transfer of DNA samples for sequencing was conducted strictly under this MTA, and no materials were used outside of the agreed scope.

### Sampling and botanical information

**Fig. 1:**
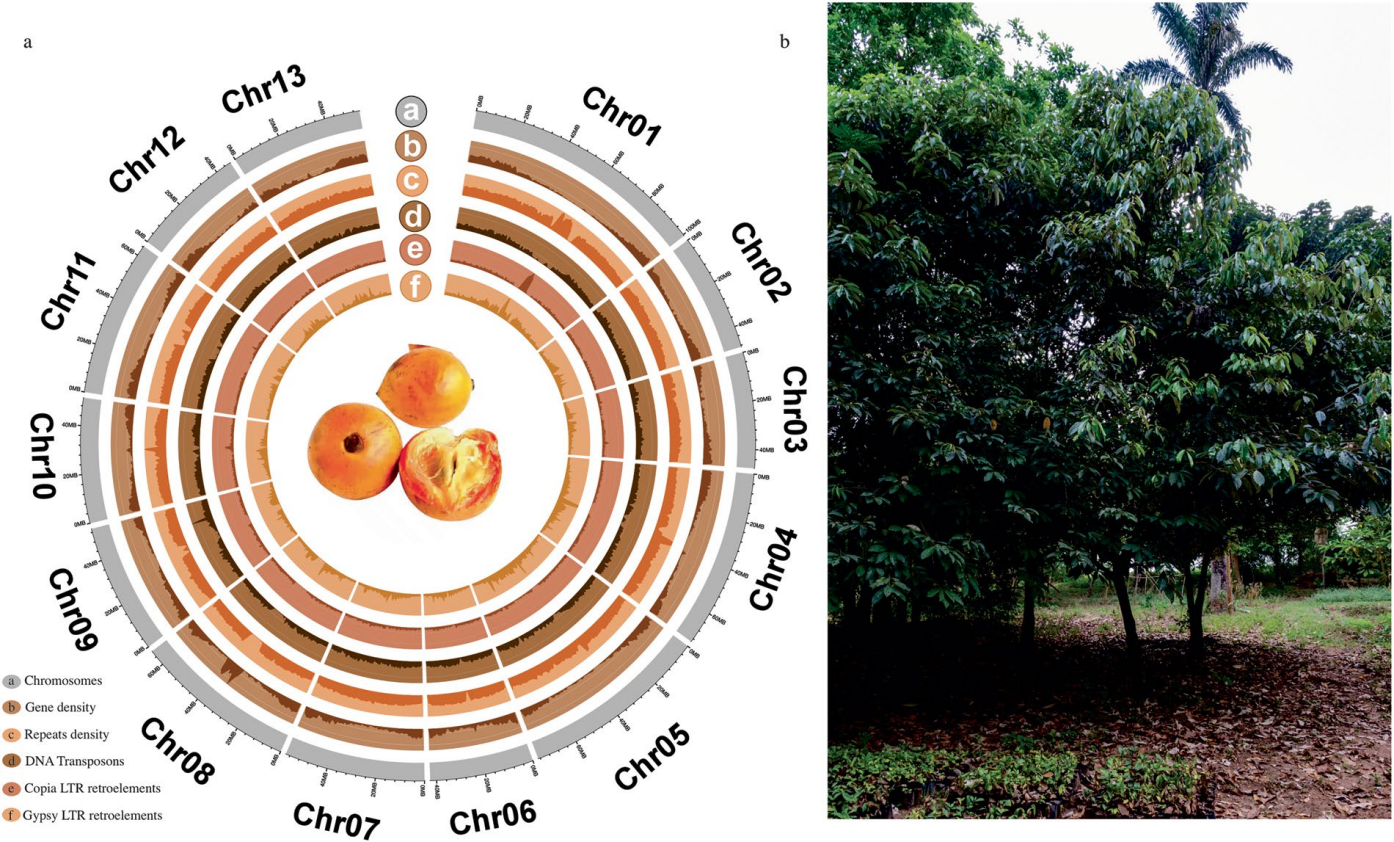
Overview of the white star apple (*Gambeya albida*) genome. (**a**) Circos plot displaying genomic features across the 13 pseudo-chromosomes calculated in 1 Mbp windows and 500 Kbp step size. From the outermost to the innermost track, the features represent: track a, the 13 assembled chromosomes (Chr01–Chr13) with tick marks indicating genomic position; track b, gene density; track c, total repeat density; track d, DNA transposon density; track e, Copia LTR retroelement density; and track f, Gypsy LTR retroelement density. (**b**) Photograph of the white star apple fruit, taken at the IITA Botanical Nursery - IITA Forest Center.

In 2012, white star apple seeds were collected from an elite mother tree, recognized for producing the sweetest fruits of the species, on the IITA campus in Ibadan, Nigeria (Fig. 1c). These seeds were sown and nurtured in the IITA Botanical Nursery in Ibadan before being planted out in the area in 2013. Among the 10 seedlings planted, this tree produced its first fruit in 2019, ahead of all others, which fruited for the first time in 2020. Leaf samples were collected from the lower branches of this tree located at N7°30.183’ E3°53.582’ (147 m above sea level). The tree measured 8.9 m in height and 15.92 cm in diameter at breast height on 27 June 2025.

### DNA Extraction and Sequencing

#### DNA Extraction

Healthy young leaves of white star apple were collected from mature trees at the IITA Botanical Nursery. Immediately after collection as described above, the leaves were placed on ice and stored at −20 °C. All the samples were processed within 24 hours of collection to ensure DNA integrity. Approximately 2.5 g of the leaves were thoroughly cleaned and finely ground into a homogeneous powder using a porcelain mortar and pestle. During grinding, liquid nitrogen was added continuously for 20 minutes to keep the sample frozen and enhance the cell lysis efficiency. After grinding, 1.5 g of the leaf powder was transferred into a pre-cooled 50 mL centrifuge tube. The homogenized sample was processed for ultra-pure, high molecular weight DNA isolation at Inqaba Biotec, Ibadan, Nigeria using the NucleoBond HMW DNA Kit (Macherey-Nagel, Germany), following the manufacturer’s instructions for chemical lysis, incubation, RNA digestion, column equilibration, binding, flushing, washing, elution, precipitation, and resuspension. The purity and concentration of the DNA were assessed using a NanoDrop spectrophotometer. DNA quality was further verified by electrophoresis on a 1% agarose gel.

#### HiFi SMRTbell Library Construction and Sequencing

The initial evaluation of the quantity and size distribution of the purified gDNA was performed using the Agilent 2200 TapeStation Nucleic Acid System (G2965AA), operated with Agilent 2200 TapeStation Software A.01.05. Analysis was conducted using the Agilent Genomic DNA ScreenTape (5067–5365) and Agilent Genomic DNA Reagents (5067–5366), with samples drawn from a 96-well plate. The isolated high molecular weight (HMW) gDNA yielded a concentration of 96 ng/µl in a total volume of 150 µl. High molecular weight genomic DNA was sheared into 15–25 kb fragments using the Megaruptor® 3 system (Diagenode, Cat# B06010003) according to the manufacturer’s specifications. An input of 66 ng/µl gDNA in a 30 µl volume was used for library preparation. HiFi SMRTbell libraries were prepared using the SMRTbell Express Template Prep Kit 2.0 (Pacific Biosciences). Sequencing was performed on two SMRT cells, with the first SMRT cell producing 1,916,709 reads, while the second generated 1,775,843 reads. The raw sequence reads were assessed for quality using the default settings of FastQC version 0.11.5 (https://www.bioinformatics.babraham.ac.uk/projects/fastqc/) to determine the parameters for the subsequent filtering step. Filtering of the raw reads was performed using Fastp version 0.23.1^27^ (parameters:–length_required 10000,–length_limit 30000, -f 15). The read length was set at an acceptable read length between 10 Kbp and 30 Kbp to enhance genome assembly performance. The filtered reads were merged, resulting in a total of 3,668,683 reads (99.36%).

#### Omni-C Library Construction and Sequencing

Frozen plant leaves (300 mg) were crushed into fine powder with a mortar and pestle in liquid nitrogen and transferred into a 50 mL Falcon tube. Formaldehyde (37% solution), PBS, and DSG were added to the ground tissue to facilitate chromatin crosslinking. After a series of washes, the enzyme mix was added to the sample for enzymatic digestion, followed by lysate QC assessment using the Tapestation D5000 kit (Agilent). The ideal fragment distribution is 100 to 2,500 bp for successful crosslinking and digestion. The next step involved performing proximity ligation by capturing the chromatin with chromatin capture beads, followed by end polishing and bridge ligation. Reverse crosslinking and DNA fragment purification were then performed. Post-DNA fragment purification and Illumina library prep (part of Dovetail Omni-C kits) were performed according to the manufacturer’s protocol. For sequencing on the PacBio Onso system, the library was converted by adding PacBio A and P1 adapter sequences in a limited PCR cycle. Sequencing was performed on the PacBio Onso system using a 300-cycle kit (2 × 150 bp), generating a total of 107,723,424 paired-end Omni-C reads. HiFi SMRTbell and Omni-C library preparation and sequencing were performed at Inqaba Biotec, Pretoria, South Africa. Quality control was conducted with FastQC (v0.11.3), and reads were filtered using fastp (v0.23.4) (parameters:–length_required 50 -f 14 -F 14). After filtering, 91,493,006 (98.5%) high-quality Omni-C reads were retained and used for genome scaffolding.

### Genome size and heterozygosity estimation

**Fig. 2:**
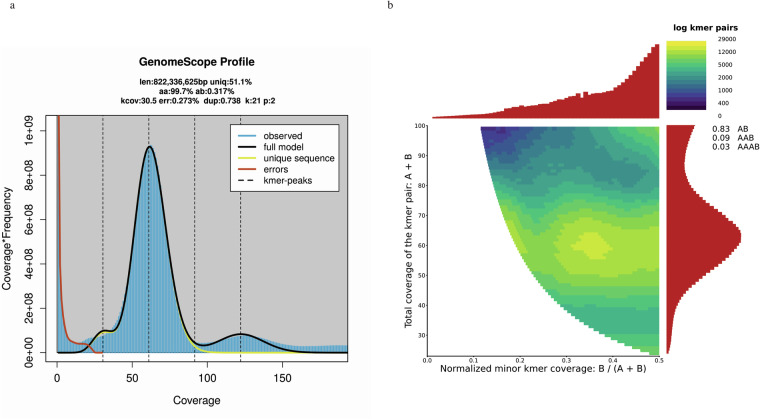
GenomeScope profile and Smudgeplot of the white star apple (*Gambeya albida*) genome. (**a**) A k-mer (21-mer) distribution curve for estimating genome size, heterozygosity, and repeat content. (**b**) Smudgeplot confirming a diploid genome (AB = 0.83).

To estimate the genome size, heterozygosity, and ploidy of the white star apple genome, k-mer analysis was performed using high-quality filtered PacBio HiFi reads. The k-mer frequency distribution was generated using Jellyfish (v2.2.8)^28^ (parameters: count -C -m 21 -s 1000000; histo). The resulting histogram was visualised with the default parameters of GenomeScope (v2.0)^29^ (Fig. 2a). The analysis estimated the haploid genome size to be approximately 822 Mbp. The k-mer distribution displays a strong unimodal peak centered around a k-mer coverage of ~60 ×, representing the homozygous portion of the genome. A minor peak near ~30 × coverage corresponds to a very small proportion of heterozygous k-mers, while the peak at ~125 × reflects repetitive or duplicated sequences. Ploidy was further assessed using Smudgeplot (v0.5.4)^29^ (Fig. 2b), which identified the AB (diploid) component as the dominant portion, accounting for 83% of k-mer pairs, with minor proportions for AAB (9%) and AAAB (3%). The low heterozygosity rate of 0.317%, combined with high homozygosity, indicates that the white star apple genome is highly homozygous.

### Genome assembly

Filtered HiFi sequence reads of approximately 75X genome coverage with an average read length of 16,951 bp were assembled *de novo* using three assembly tools. Hifiasm v0.16.1-r375 (parameter:–primary), HiCanu v2.3 (parameters: -pacbio-hifi genomeSize = 800 m -useGrid = false -merylThreads = 4 -merylMemory = 8 corOverlapper = ovl), and Flye v2.9.6-b1802 (parameters:–pacbio-hifi–genome-size 800 m)^30–32^. The genome statistics from the three assemblies were compared using the default settings of QUAST^33^ (v5.1.0rc1) (Table 1).

**Table 1:** Genome assembly quality metrics for different assemblies.

	HiFiasm assembly	HiCanu assembly	Flye assembly	HiFiasm assembly purged	HiFiasm assembly purged (second round)	Hi-C scaffolded assembly	Final assembly
**Total length (Mbp)**	2407.39	2832.63	1532.66	901.47	821.34	821.38	813.97
**Number of contig/scaffold**	7111	28200	26505	1114	1023	596	586
**N50 (Mbp)**	1.36	0.24	0.11	3.01	3.11	57.68	57.68
**L50**	454	1707	3420	95	84	6	6
**N90 (Mbp)**	0.15	0.04	0.03	0.68	0.78	30.45	46.68
**L90**	2353	16719	14831	322	280	13	12
**Gaps**	0	0	0	0	0	1153	146

On the basis of the N50 value and the number of contigs, we selected the hifiasm primary assembly. This assembly had an N50 of 1.36 Mbp and the fewest number of contigs (7,111), making it the most contiguous draft genome among the outputs of the three assemblers. However, the total size of the draft genome was larger than its estimated genome size, likely due to a high level of duplication (87.6%) (Table 3). We implemented a two-round purging strategy using the purge_dups pipeline^34^. First, PacBio clean HiFi reads were aligned to the draft primary assembly using Minimap2 (v2.24-r1122)^35^, and coverage statistics along with cutoff thresholds were computed to inform the purging process. The assembly was then split and self-aligned with Minimap2 to identify and remove redundant haplotigs. The final purged genome (draft genome) had an N50 of 3.1 Mbp and a total size of 821 Mbp and was subsequently used for scaffolding.

### Omni-C scaffolding, manual curation, and gap filling

A chromosome-scale assembly comprising thirteen chromosomes was constructed by integrating Omni-C data with the contig-level draft assembly, followed by manual curation and gap filling (Fig. 1a). Filtered high-quality paired-end Omni-C reads were aligned to the draft genome using bwa (v0.7.19-r1273)^36^ (parameters: mem -SP -T0). The resulting alignments were processed using the pairtools (v1.1.2)^37^, which performed parsing, sorting, duplicate marking, and splitting of read pairs to generate a contact map. The final alignment file was sorted and indexed using samtools (v1.15.1)^38^ and was used for scaffolding with YaHS (v1.2.2)^39^ with default parameters, which incorporated the proximity ligation data to reorder and orient contigs into scaffolds. YaHS’s alignment matrix was subsequently converted into a contact map in pairs format using PretextMap (https://github.com/sanger-tol/PretextMap) (parameters:–sortby length–sortorder descend–mapq 0), which was then visualized in PretextView (https://github.com/sanger-tol/PretextView) for manual curation. The PretextView enabled inspection of Omni-C read contact patterns against the scaffolds, allowing correction of misassemblies and refinement of scaffold anchoring and orientation into chromosome-scale pseudomolecules, guided by the genomic proximity signal of the Omni-C data.

To reduce assembly fragmentation, gaps introduced during scaffolding and manual curation were filled using LR_GapCloser^40^. LR_Gapcloser employs a tiling path-based approach by fragmenting long reads into short tags and aligning them using bwa (v0.7.19-r1273) to the assembly to identify and fill gaps. Before gap filling, the curated genome contained 1,153 gaps. Using default parameters, LR_Gapcloser reduced the number of gaps to 146. The final chromosome-scaled genome had an improved contiguity of N50 value of 57 Mbp and an L90 value of 12 (Table 1). The assembly was validated using Omni-C contact data. The genome-wide contact map generated with PretextMap and visualized in PretextView (v1.0.7) (Fig. S1).

#### Transposable Elements (TE) annotation

**Fig. 3:**
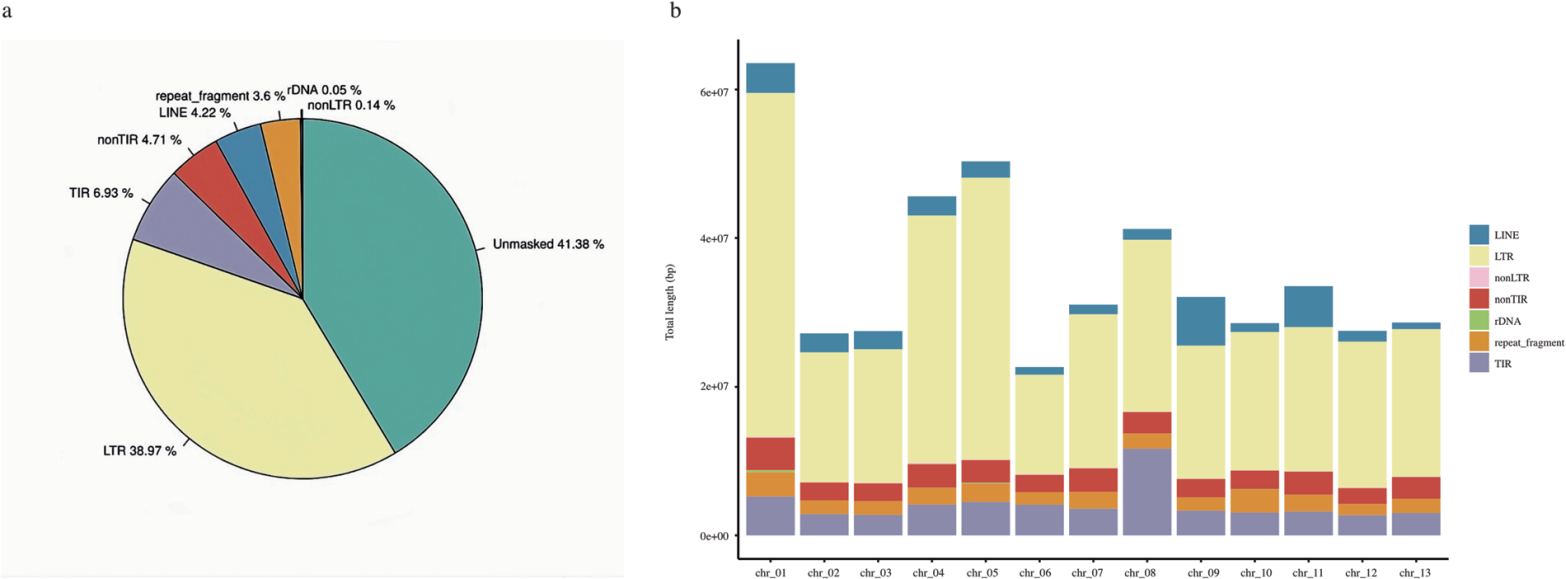
Proportion and distribution of transposable elements (TEs) across the chromosomes of the white star apple (*Gambeya albida*) genome (**a**) Proportion of the percentage TEs covering the genome (**b**) Distribution of TEs across all chromosomes.

The repeat landscape of *G. albida* was characterized and annotated using Extensive *de novo* TE Annotator (EDTA) v2.2.2^41^ (parameters: –genome –overwrite 1 –sensitive 1 –anno 1 –evaluate 1). This pipeline integrates structure-based and homology-based approaches to detect primary TEs within the assembled genome. It employs a combination of specialized tools, including HelitronScanner, LTR_FINDER, LTRHarvest, LTR_retriever, TIR-Learner, RepeatModeler2, and RepeatMasker, to classify and annotate novel TE sequences^42–47^. The repeats accounted for 58.62% of the genome. EDTA was able to categorise the repeats into major categories such as LTR transposons (38.97%), LINE transposons (4.22%), TIR (6.93%), non-TIR (4,71%), non-LTR (0.41%), and other repeats (Fig. 3a). LTR transposons were the most abundant repeats identified across the genome (Fig. 3b). After repeat identification and annotation, the genome was soft-masked using the *make_masked.pl* utility from the EDTA pipeline, and the masked genome was subsequently used for gene prediction.

#### Gene Prediction and Functional Annotation

Protein-coding gene models were predicted using Helixer (v0.3.6)^48^, a deep-learning-based *ab initio* gene predictor that employs a pre-trained, lineage-specific neural network to infer gene structure directly from genomic sequence. The “land_plant” model was applied to the final chromosome-scale assembly, enabling gene prediction in the absence of RNA sequencing data and yielding 33,602 protein-coding genes (Table 2). Corresponding protein sequences were extracted from the predicted gene models using gffread^49^. Functional annotation of the predicted proteome was performed using a combination of homology and domain-based approaches. Orthology assignments and functional descriptors were obtained by querying the eggNOG database (v5.0.2) with eggNOG-mapper (v2.1.12)^50^ (parameters:–decorate_gff and -m diamond). Protein domain and family annotations were derived from the InterPro member databases^51–53^ using InterProScan (v5.60-92.0) (parameters: -appl Pfam, PANTHER, Gene3D, TIGRFAM, CDD). In parallel, predicted proteins were aligned against the UniProt/Swiss-Prot database^54^ (Last modified: 2026-06-10) using BLASTP (v2.6.0 +) (parameters: -outfmt 6, -evalue 1e-6, -max_target_seqs. 1). BLASTP alignments were filtered, and only hits with ≥ 40% sequence identity and an alignment length of more than 100 amino acids were parsed into the final annotation.

**Table 2:** Annotation counts and gene models supported with functional annotation.

Feature	Count
**Genes**	33,602
**CDS**	33,602
**Total exons**	213208
**Number of genes with CDD annotations**	10,886
**Number of genes with Gene3D annotations**	21,116
**Number of genes with InterProScan annotations**	27,165
**Number of genes with PANTHER annotations**	28,768
**Number of genes with Pfam annotations**	25,152
**Number of genes with TIGRFAM annotations**	2,833

## Data Records

The raw PacBio HiFi reads and Omni-C reads used for the assembly are available from the National Center for Biotechnology Information (NCBI) Sequence Read Archive (SRA) database under the BioProject PRJNA1255872 with accession numbers SRR33323859^55^ and SRR33422445^56^, respectively. The genome has been submitted under the same BioProject with the accession number JBNXVW000000000^57^. The genome assembly FASTA file and annotation GFF3 files are uploaded to Zenodo^58^ (10.5281/zenodo.20794479).

## Technical Validation

To assess the accuracy and completeness of the white star apple genome assembly, we conducted a Benchmarking Universal Single-Copy Orthologs (BUSCO)^59^ analysis using the eudicots lineage dataset on both the draft and final assemblies. This analysis identified 2,267 complete BUSCO genes out of 2,326, indicating a genome completeness of 97.5%. The proportion of duplicated BUSCOs decreased from 87.6% in the draft assembly to 6.1% in the final assembly, reflecting the successful removal of redundant haplotypic sequences during assembly refinement (Table 3). We aligned clean PacBio HiFi reads to the final assembly using Minimap2 to evaluate assembly accuracy, yielding a mapping rate of 99.29%. Additionally, a *k-mer-*based quality assessment using merqury^60^ (v1.3) (*k-mer* = 21) produced a quality value (QV) score of 58.14 and a k-mer completeness of 88.10% (Table 4). To further evaluate the completeness of the predicted gene set, we performed a complementary BUSCO analysis on the annotated proteome using the embryophyta lineage dataset, recovering 92.5% complete BUSCOs (Table 3). Together, these assessments confirmed the high quality and completeness of both the white star apple genome assembly and its gene annotation.

**Table 3:** BUSCO assessment of the *G.albida* genome assembly and gene annotation.

	Draft assembly		Final assembly		Predicted protein	
	Number	Percent (%)	Number	Percent (%)	Number	Percent (%)
**Complete BUSCOs (C)**	2296	98.7%	2267	97.5%	2326	92.4%
**Complete and single-copy BUSCOs (S)**	258	11.1%	2124	91.3%	2007	86.3%
**Complete and duplicated BUSCOs (D)**	2038	87.6%	143	6.1%	143	6.1%
**Fragmented BUSCOs (F)**	9	0.4%	8	0.3%	96	4.1%
**Missing BUSCOs (M)**	21	0.9%	51	2.2%	80	3.5%
**Total BUSCO groups searched**	2326		2326		2326

**Table 4:** Quality value scores of the *G.albida* genome assembly.

	Quality Value (QV) score	K-mer completeness
**White star apple genome**	58.1437	88.1048

## Supplementary information

Fig. S1

## Data Availability

All bioinformatic analyses were performed using publicly available software, following the parameters and protocols described in the Methods. No custom scripts were required. All tools were used with their default parameters unless otherwise stated. The specific commands and parameters used for each step of the analysis are available at https://github.com/LandiMi2/StarAppleGenomeAnalysis/tree/main.
